# Thrombotic microangiopathy with transiently positive direct Coombs test in an adult with poststreptococcal acute glomerulonephritis: a case report

**DOI:** 10.1186/s12882-022-02684-z

**Published:** 2022-02-05

**Authors:** Dan Inoue, Takashi Oda, Sachiko Iwama, Takahiko Hoshino, Mitsuya Mukae, Takashi Sakai, Aki Kojima, Takahiro Uchida, Tadasu Kojima, Kentaro Sugisaki, Tomohiro Tomiyasu, Noriko Yoshikawa, Muneharu Yamada

**Affiliations:** Department of Nephrology and Blood Purification, Kidney Disease Center, Tokyo Medical University Hachioji Medical Center, 1163 Tatemachi, Hachioji, Tokyo, 193-0998 Japan

**Keywords:** Poststreptococcal acute glomerulonephritis (PSAGN), Thrombotic microangiopathy (TMA), Direct Coombs test, Neuraminidase, Thomsen–Friedenreich (T) antigen, Nephritis-associated plasmin receptor (NAPlr), Case report

## Abstract

**Background:**

To date, a few case reports have described the association between poststreptococcal acute glomerulonephritis (PSAGN) and hemolytic anemia/thrombocytopenia, both with or without a pathology similar to that of thrombotic microangiopathy (TMA). However, the detailed mechanism leading to the complication of TMA in PSAGN patients remains to be clarified. In contrast, infection with neuraminidase-producing *Streptococcus pneumoniae* is a well-known cause of TMA, and it has been reported that transient positivity of the direct Coombs test is observed in up to 90% of such patients.

**Case presentation:**

A 44-year-old man was hospitalized for acute nephritic syndrome 3 weeks after developing pharyngitis. PSAGN was suspected owing to a low complement C3, increased antistreptolysin-O and serum creatinine (5.46 mg/dL), and hematuria/proteinuria. The throat antigen test for group A *Streptococcus* was positive. He developed hemolytic anemia with thrombocytopenia from hospital day 9. TMA was suspected owing to minimal coagulation abnormalities. ADAMTS-13 activity was normal, whereas the direct Coombs test was transiently positive. Renal biopsy demonstrated glomerular endocapillary proliferation without crescents, but with severe tubulitis and peritubular capillaritis on light microscopy. Immunofluorescence demonstrated C3 deposition along the glomerular capillary walls, and many subepithelial humps were observed on electron microscopy. The deposition of nephritis-associated plasmin receptor (NAPlr), a nephritogenic protein of *Streptococcus pyogenes*, was observed only in glomeruli. Thus, the histological diagnosis was typical PSAGN, but with atypical severe tubulointerstitial lesions. A positive direct Coombs test is often observed in pneumococcal TMA patients, which is attributed to the exposure of Thomsen–Friedenreich (T) antigen by neuraminidase. As *Streptococcus pyogenes* is one of the neuraminidase-producing bacteria other than *Streptococcus pneumoniae*, T-antigen exposure was analyzed in the renal tissue of this patient using labelled peanut lectin as a probe, which has strong and specific binding affinity for T-antigen. Exposure of T-antigen was found on tubular epithelial cells and small vessels in the tubulointerstitial area, but not in the glomeruli of this patient.

**Conclusion:**

These findings suggest that 2 pathogenic proteins of *Streptococcus pyogenes*, i.e., NAPlr and neuraminidase, induced glomerular lesions of PSAGN and tubulointerstitial inflammation with TMA, respectively, resulting in severe acute kidney injury in this patient.

## Background

Poststreptococcal acute glomerulonephritis (PSAGN) is a disease that develops following streptococcal infection, with a latent period of more than a week. It is known as the most common cause of pediatric acute glomerulonephritis with a favorable prognosis; however, the clinical manifestations of PSAGN in adults are often more severe, and develop into chronicity with an unfavorable prognosis [1, 2].

Anemia is often observed in patients with PSAGN, and there have been a few case reports describing the association of PSAGN with hemolytic anemia and thrombocytopenia, both with or without a pathology similar to that of thrombotic microangiopathy (TMA) [3–6]. However, the detailed mechanism leading to the complication of TMA in PSAGN patients remains to be clarified. On the other hand, infection with neuraminidase-producing *Streptococcus pneumoniae* is a well-known cause of TMA [7]. Interestingly, a unique clinical manifestation, namely, transient positivity for the direct Coombs test [8, 9], is observed in up to 90% of these patients. Exposure of the hidden Thomsen–Friedenreich (T) antigen via bacterial neuraminidase has been suspected as the pathogenic mechanism of TMA in such patients [10, 11]. Pneumococcal neuraminidase exposes the T-antigen transiently on the surface of erythrocytes, platelets, and endothelial cells. Naturally existing preformed host immunoglobulin (Ig) M antibodies in the plasma of the infected host bind to the exposed T-antigen, resulting in vascular endothelial injury, which can initiate a cascade of events leading to TMA.

We here describe a case of a patient with PSAGN complicated by TMA, whose direct Coombs test was transiently positive. Pathological analysis demonstrated glomerular changes typical of PSAGN with glomerular deposition of nephritis-associated plasmin receptor (NAPlr), a nephritogenic protein of *Streptococcus pyogenes*, but also atypical severe tubulointerstitial changes accompanied by the exposure of T-antigens on the tubules and vascular endothelial cells.

## Case presentation

A 44-year-old Japanese man with well-controlled hypertension, and without any history of renal dysfunction or urinary abnormalities presented with a fever and pharyngeal pain. He was first examined at a local clinic, and was prescribed antibiotics for 10 days. He reported facial and leg edema 19 days after the consultation, and was admitted to our hospital. He has been treated for sleep apnea syndrome over the past two years, but had no family history of kidney disease.

On admission, the patient’s body temperature was 36.9 °C, and his blood pressure was 175/109 mmHg. He had edema around his eyes and feet. A chest X-ray, electrocardiography, and abdominal ultrasound were performed, but did not show any apparent abnormalities.

The patient’s initial laboratory test results are summarized in Table 1. He showed nephritic urinary abnormalities and severe kidney failure (urinary protein 3+, urinary occult blood 3+, serum creatinine 5.46 mg/dL). His CRP (4.05 mg/dL), antistreptolysin-O titer (3440 IU/mL), antistreptokinase titer (40,960 IU/mL) and gamma globulins (IgG/IgA/IgM: 2130/660/50 mg/dL) were increased. His complement C3 level was low (28.8 mg/dL), while C4 level was normal. Autoantibodies, such as the antinuclear antibody, anti–double-stranded DNA antibody, and antineutrophil cytoplasmic antibody were undetectable. The rapid antigen test on a throat swab sample demonstrated positivity for group A beta-hemolytic *Streptococcus* (*Streptococcus pyogenes*). Urinary pneumococcal (*Streptococcus pneumoniae*) antigen was negative. Thus, the patient had developed acute nephritic syndrome with severe renal insufficiency following an antecedent pharyngitis with *Streptococcus pyogenes* after a latent period of approximately 3 weeks, and a clinical diagnosis of severe PSAGN was made.

**Table 1:** Laboratory data at the time of admission

Urinalysis		Biochemistry, coagulation, immunological, and bacteriologic parameters			
specific gravity	1.015	TP	8.2 g/dL	ASO	3440 IU/mL
pH	5.0	Alb	3.5 g/dL	ASK	40960 IU/mL
protein	3+	BUN	17.5 mg/dL	IgG	2130 mg/dL
glucose	–	Cr	5.46 mg/dL	IgA	660 mg/dL
occult blood	3+	AST	24 IU/L	IgM	50 mg/dL
Red blood cells	20–29/HPF	ALT	28 IU/L	C3	28.8 mg/dL
White blood cells	30–49/HPF	T-bil	0.7 mg/dL	C4	27.9 mg/dL
**Complete blood cell count**		LDH	329 IU/L	CH50	35.6 U/mL
White blood cells	14.2× 10^3^/μ	CK	121 IU/L	ANA	< 40
Neutrophils	78.3%	Na	137 mEq/L	MPO-ANCA	< 1.0 U/mL
Eosinophils	1.8%	K	4.6 mEq/L	PR3-ANCA	< 1.0 U/mL
Basophils	0.1%	Cl	101 mEq/L		
Monocytes	9.6%	Ca	8.1 mg/dL		
Lymphocytes	10.1%	IP	6.3 mg/dL		
Red blood cells	4.25 × 10^6^/μ	CRP	4.05 mg/dL	A rapid antigen detection test for GAS	positive
Hemoglobin	13.6 g/dL	FDP	78.3 μg/mL		
Hematocrit	40.0%	PT-INR	1.06		
Platelets	13.5 × 10^4^/μL	APTT/cont.	49.9/33.6 s		
		Fibrinogen	563 mg/dL		

*TP* total protein, *Alb* albumin, *BUN* blood urea nitrogen, *Cr* creatinine, *AST* aspartate transaminase, *ALT* alanine transaminase, *T-bil* total bilirubin*, LDH* lactate dehydrogenase, *CK* creatine kinase, *CRP* C-reactive protein, *FDP* fibrin degradation products, *PT* prothrombin time, *APTT* activated partial thromboplastin time, *ASO* anti-streptolysin O, *ASK* anti-streptokinase, *IgG* Immunoglobulin G, *IgA* Immunoglobulin A, *IgM* Immunoglobulin M, *C3* Complement 3, *C4* Complement 4, *CH50* 50% hemolytic complement activity, *ANA* antinuclear antibody, *ANCA* anti-neutrophil cytoplasmic antibody, *GAS Group A beta-hemolytic streptococcus*

**Fig. 1:**
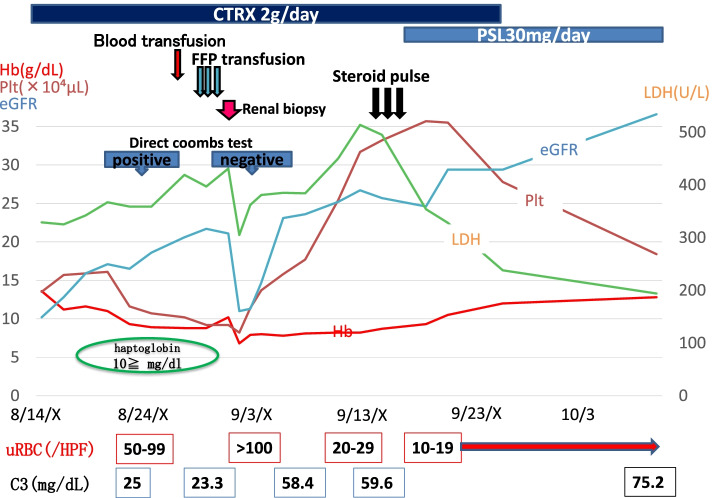
Summary of the clinical course of the patient showing a continuous improving trend. The complication of thrombotic microangiopathy (TMA) with a transiently positive direct Coombs test was observed. A renal biopsy was performed on hospital day 18. The patient was discharged from the hospital on hospital day 44, after his serum creatinine level recovered to 2.0 mg/dL. In general, all clinical data (such as renal function, urinalysis abnormalities, and serum C3 level) showed a continuously improving trend

The patient’s clinical course is summarized in Fig. 1. Symptomatic treatment was initially performed, but as the patient had a mild fever and high CRP level, 2 g of ceftriaxone was administered every 24 h in consideration of a possible latent infection. Although the patient’s kidney function gradually improved, the severe hematuria continued. Moreover, his hemoglobin level and platelet count decreased. Hemolytic anemia was suspected because his haptoglobin level was less than 10 mg/dL, and LDH was high at 514 IU/L. Hemolytic anemia (Hb: 8.8 g/dL), thrombocytopenia (Plt: 9.2 × 10^4^ μL), and severe renal failure, with minimal abnormalities in coagulation tests suggested a complication of TMA. The plasma level of ADAMTS-13 activity was normal (88%). Stool culture and assays for Shiga-like toxin and an O157 LPS antibody, respectively, were negative. Thus, thrombotic thrombocytopenic purpura and Shiga toxin-producing *Escherichia coli*-associated hemolytic uremic syndrome (HUS) were unlikely. Meanwhile, the direct Coombs test was transiently positive, i.e., it was positive when performed on hospital day 11, whereas that performed on hospital day 21 was negative. A renal biopsy was performed after improvement in the patient’s anemia and thrombocytopenia, after a blood and fresh frozen plasma transfusion on hospital day 18.

**Fig. 2:**
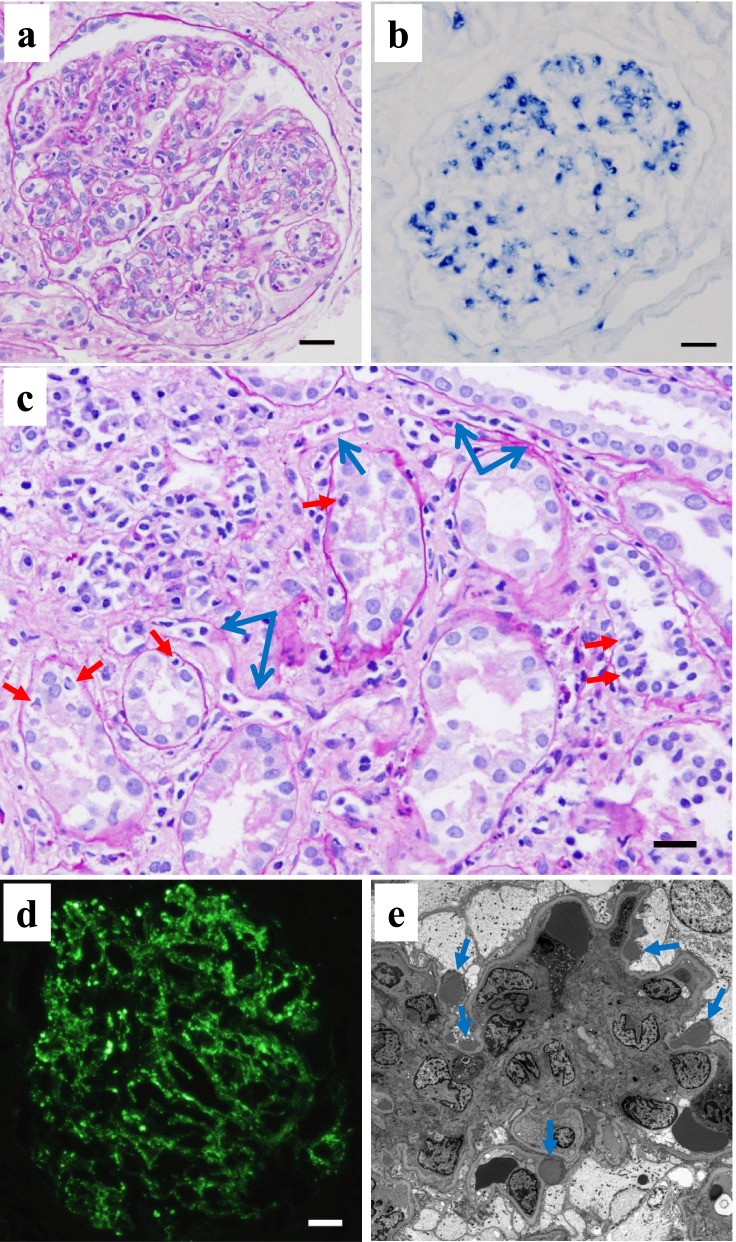
Histological features of the renal biopsy showing the endocapillary proliferative glomerulonephritis typical of poststreptococcal acute glomerulonephritis (PSAGN) but with atypical severe tubulointerstitial change. **a** Periodic acid-Schiff (PAS) staining of formalin-fixed paraffin-embedded tissue (FFPE) sections. Diffuse global endocapillary proliferation with massive infiltration of neutrophils was observed (Scale bar = 20.0 μm). **b** Chloroesterase staining, which detects neutrophils, confirmed the massive accumulation of neutrophils in glomeruli (Scale bar = 20.0 μm). **c** Tubulointerstitial lesions with marked infiltration of inflammatory cells were observed. Red arrows indicate the regions of tubulitis, and blue arrows indicate the regions of capillaritis (PAS, Scale bar = 20.0 μm). **d** Immunofluorescence (IF) microscopy showed granular deposition of C3 mainly along the glomerular capillary walls (Scale bar = 20.0 μm). **e** Electron microscopy showed many subepithelial electron dense deposits (indicated by blue arrows) with a hump-shaped appearance

Light microscopy analysis of formalin fixed paraffin embedded tissue (FFPE) sections of renal biopsy containing 11 glomeruli demonstrated no sclerosis or crescents, but diffuse and global glomerular endocapillary proliferation with massive infiltration of neutrophils was observed (Fig. 2a). The glomerular massive infiltration of neutrophils was further confirmed by chloroesterase staining (Fig. 2b). On the other hand, marked infiltration of inflammatory cells (consisted mainly of mononuclear cells without eosinophils) in the tubulointerstitial area, with tubulitis, capillaritis, and interstitial inflammation was observed (Fig. 2c). Immunofluorescence analysis of fresh frozen tissue sections demonstrated strong glomerular C3 deposition along the capillary walls (Fig. 2d), but no immunoglobulin or C1q deposition (data not shown). Electron microscopy showed many subepithelial humps (Fig. 2e).

**Fig. 3:**
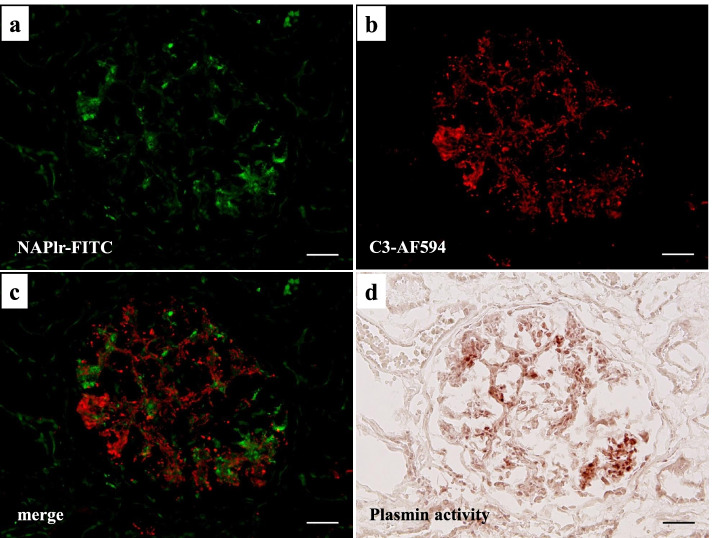
Evaluation of glomerular damage by histological staining of fresh frozen tissue sections showing the positive glomerular staining of nephritis-associated plasmin receptor (NAPlr) and plasmin activity in simillar fashion, supporting the diagnosis of PSAGN. **a** NAPlr was positive by IF staining, mainly in the inner side of glomerular tufts (fluorescein isothiocyanate [FITC], green). **b** C3 staining on the same section was distributed mainly along the capillary walls (Alexa Fluor 594 [AF594], red). **c** A merged image demonstrating the different localizations of NAPlr and C3. **d** In situ zymography for plasmin activity in an adjacent section to (**a**) showed positive staining in the glomerulus with a similar distribution to NAPlr staining. Scale bars = 20.0 μm in (**a**)-(**d**)

**Fig. 4:**
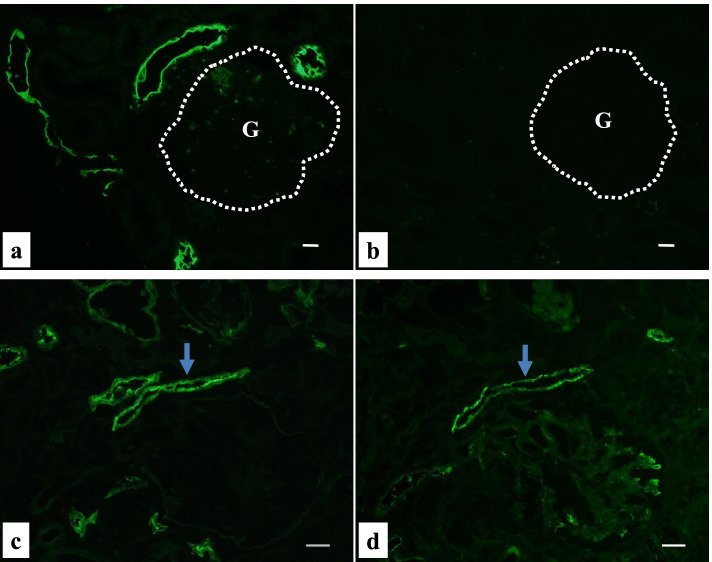
Histological evaluation of Thomsen–Friedenreich (T) antigen exposure in renal biopsy tissue showing its exposure in tubointerstitial lesions, and possible its interaction with IgM. **a** Staining with FITC-conjugated peanut lectin of a FFPE section from this patient demonstrated specific binding of peanut lectin to some tubular epithelial cells and some vascular endothelial cells, suggesting T-antigen exposure on these cells, whereas binding was not observed in a glomerulus (indicated as G). **b** The same staining of a FFPE section from a normal control showed negative results. A glomerulus was indicated as G. (**c, d**) Relative distribution of T-antigen exposure and IgM deposition. **c** Staining of fresh-frozen tissue with FITC-conjugated peanut lectin revealed positive staining of vascular endothelial cells adjacent to a glomerulus. **d** IF staining for IgM in an adjacent section to (**c**) revealed the deposition of IgM at the T-antigen-exposed site (indicated by blue arrows in **c** and **d**). Scale bars = 20.0 μm in (**a**)-(**d**)

**Fig. 5:**
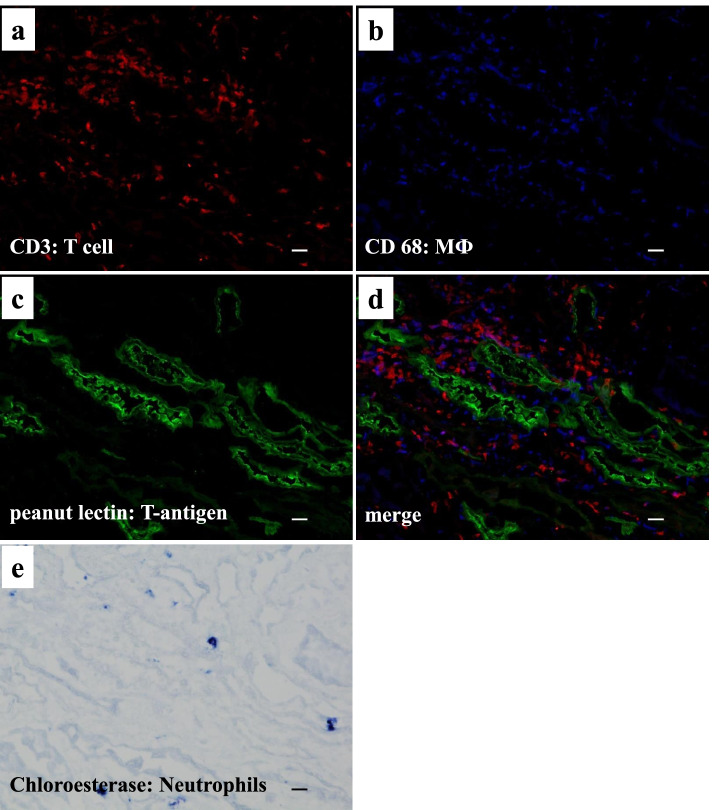
Multiple staining for CD3, CD68, T-antigen, and neutrophils on a fresh frozen biopsy section showing the relationship between T-antigen exposed site and the inflammatory cells infiltration. **a** Indirect IF staining for CD3 (Alexa Fluor 594: red) and (**b**) CD68 (Alexa Fluor 350: blue), (**c**) direct staining with FITC-conjugated peanut lectin (T-antigen: green), and (**e**) chloroesterase staining (light microscopy, blue) were performed on a section of fresh frozen renal tissue from the patient. A merged image of the staining results (**d**) revealed the massive accumulation of CD3-positive T-cells and CD68-positive macrophages around the peanut lectin-positive tubulointerstitial lesions, although infiltration of chloroesterase-positive neutrophils was rare (**e**). Scale bars = 20.0 μm in (**a**)-(**e**)

We also analyzed the presence of NAPlr, a nephritogenic protein isolated from *Streptococcus pyogenes*, and performed in situ zymography for plasmin activity on serial sections of fresh frozen tissue. NAPlr deposition and associated plasmin activity were observed in glomeruli with a similar staining pattern (Fig. 3a-d), supporting the histological diagnosis of PSAGN [12]. Thus, histologically, glomerular lesions of this patient were typical of PSAGN. On the other hand, the severe tubulointerstitial changes that were observed are not the usual histological findings of PSAGN. Therefore, to evaluate the pathogenesis of TMA in a patient with a transiently positive direct Coombs test, we analyzed the FFPE sections of renal biopsy with fluorescein isothiocyanate-labelled peanut lectin (Thermo Fisher Scientific, Waltham, MA), which is known to have strong and specific binding affinity for T-antigen [13, 14]. We found the specific binding of peanut lectin to some tubular epithelial cells and vascular endothelial cells in the cortical tubulointerstitial area, whereas such binding was not observed in glomeruli in the same field (Fig. 4a) or in a similar area of a normal control kidney (Fig. 4b). Furthermore, staining for IgM and T-antigen on serial sections of fresh frozen renal biopsy tissue demonstrated IgM deposition in the T-antigen-exposed sites (Fig. 4c, d). In addition, multiple staining for CD3, CD68, peanut lectin, and chloroesterase (a neutrophil marker) on fresh frozen tissue sections revealed massive accumulation of CD3-positive T cells and CD68-positive macrophages around the peanut lectin-positive tubulointerstitial lesions. In contrast, there was minimal infiltration of neutrophils in these area (Fig. 5 a–e).

The patient began steroid pulse therapy with methylprednisolone (500 mg/day for 3 days) from hospital day 31, followed by oral prednisolone (30 mg/day) before the renal biopsy results were confirmed, because the patient demonstrated severe renal insufficiency and severe hematuria even though active infection was absent and his platelet count had improved. He was well-tolerated for the treatment with few side effects. His serum creatinine level recovered to 2.0 mg/dL. He was discharged from the hospital on day 44 and was followed-up as an outpatient. His complement levels became normal 13 weeks after the onset of symptoms, and his serum creatinine was 1.15 mg/dL with no hematuria or proteinuria at the most recent follow-up.

## Discussion and conclusions

Infection with neuraminidase-producing bacteria, of which *Streptococcus pneumoniae* is a representative example, has been reported to lead to TMA via the following mechanism. T-antigens, which are normally hidden by neuraminic acid, are exposed transiently on the surface of erythrocytes, platelets, and endothelial cells by bacterial neuraminidase. Preformed IgM antibodies of the infected host bind to the exposed T-antigen, resulting in vascular endothelial injury, which can initiate a cascade of events leading to TMA [11]. Interestingly, the direct Coombs test, which detects antibodies adhering to the surface of red blood cells, is transiently positive in up to 90% of pneumococcal TMA patients [8, 9]. TMA with a transiently positive direct Coombs test strongly suggests the involvement of such events in this patient. However, the pathogenic bacterium in this patient was not *Streptococcus pneumoniae* but was *Streptococcus pyogenes* based on the following findings: negative result on urinary pneumococcal antigen test and positive result on rapid antigen Group A streptococcus test, which has high sensitivity and specificity for *Streptococcus pyogenes* in adults [15]. Several pathogens are known to produce neuraminidase, and *Streptococcus pyogenes* is another neuraminidase-producing bacteria reported to cause TMA via a similar mechanism [16, 17]. Indeed, we demonstrated the exposure of T-antigens in the renal tubules and small vessels of this patient using peanut lectin, which has high affinity and specificity for the T-antigen, and therefore, is commonly used as a probe for detecting T-antigen exposure [13, 14]. In contrast to the report of Shimizu et al. [18], which demonstrated T-antigen exposure in the glomeruli as well as in the tubules of a patient with *Streptococcus pyogenes*-associated hemolytic uremic syndrome, we found the exposed T-antigens only in the tubulointerstitial area, more specifically on tubular epithelial cells and on vascular endothelial cells, but none in the glomeruli. The distinct reason for the incomplete T-antigen exposure in this case is not known for now, however, cases with similar histological findings, i.e., exposure of T-antigens only in the tubulointerstitial area without glomeruli have been reported in patients with TMA associated with *Capnocytophaga* infection [19] as well as *Streptococcus pneumoniae infection* [10]. On the other hand, a recent study reported that galectin-3, a T-antigen-binding protein, interacts with T-antigen and contributes to the development of pneumococcal HUS [20]. Thus, T-antigen exposure may be associated with various bacterial and host related factors such as bacterial neuraminidase producing activity and galectin-3 concentration in the host, and therefore the entire picture of precise mechanism of T-antigen exposure related TMA needs to be clarified in the future.

As to the glomerular lesions of this patient, the diagnosis of PSAGN was quite clear both clinically and histologically, and was supported by the positive glomerular staining for NAPlr and plasmin activity [12]. Staining for NAPlr and plasmin activity was negative in the tubulointerstitial area. Similarly, neutrophils assessed by chloroesterase staining were prominently accumulated in glomeruli, but did not accumulate in tubulointerstitial lesions. Thus, the tubulointerstitial lesions in this patient seemed to be developed through the mechanism different from glomerular lesions. We therefore performed the indirect immunofluorescent staining for streptococcal pyrogenic exotoxin B (SPE B), another potential nephritogenic antigen for PSAGN and is reported to induce tubulointerstitial nephritis with its deposition [21]. SPE B, however, was not observed at all in the tubulointerstitial area (data not shown), so the possibility of SPE B induced tubulointerstitial nephritis was unlikely. Another possibility is the superimposition of drug-induced tubulointerstitial nephritis. Although it is impossible to completely exclude this possibility, the clinical manifestations indicating drug allergy, such as eosinophilia and liver dysfunction were not observed in this patient (Table 1), and tubulointerstitial infiltration of eosinophils, histological characteristic of drug-induced tubulointerstitial nephritis, was not observed either. And above all, close relationship between T-antigen exposed site and the inflammatory cells infiltration shown by the results of multiple staining for CD3, CD68, and peanut lectin, strongly suggested that T-antigen exposure by neuraminidase of *Streptococcus pyogenes* might be involved in the tubulointerstitial inflammation of this patient.

**Fig. 6:**
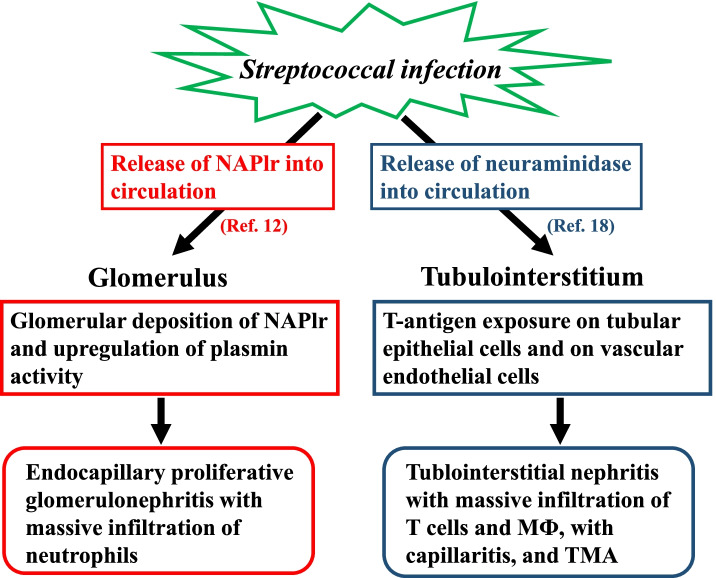
Putative etiology of the pathological condition of this patient summarizing 2 toxic agents of *Streptococcal pyogenes* locally induce inflammation in the glomeruli and tubulointerstitium in different ways. Infection with *Streptococcal pyogenes*, which produces the nephritis-associated plasmin receptor (NAPlr) and neuraminidase, induces the release of NAPlr and neuraminidase into the circulation. On one hand, circulating NAPlr accumulates on the inner side of the glomerular tufts, and then traps and maintains the activity of plasmin, which induces plasmin-associated glomerular damage leading to the development of endocapillary proliferative glomerulonephritis with massive infiltration of neutrophils (Ref. 12). On the other hand, the circulating neuraminidase (Ref. 18) affects renal tubular epithelial cells, vascular endothelial cells, and red blood cells, leading to the exposure of the T-antigen on these cells, resulting in the development of tubulointerstitial nephritis with T-cells and macrophage (MФ) infiltration, capillaritis, and thrombotic microangiopathy (TMA). Thus, the 2 toxic agents of *Streptococcal pyogenes* locally induce inflammation in the glomeruli and tubulointerstitium in different ways, resulting in the accumulation of different cell populations in the two regions

From these findings, we suspect that two pathogenic proteins produced in *Streptococcus pyogenes*, i.e., NAPlr and neuraminidase, play important roles in the development of renal damage in different regions of the kidney (Fig. 6). The glomerular manifestation of PSAGN such as neutrophil accumulation was induced essentially by the glomerular deposition of NAPlr and associated plasmin activity. On the other hand, neuraminidase activity of *Streptococcus pyogenes* results in exposure of the T-antigen on tubular epithelial cells and vascular endothelial cells in the tubulointerstitial area, leading to the development of tubulointerstitial inflammation with T cells and macrophages and Coombs test-positive TMA. The involvement of both mechanisms is thought to have caused the severe acute kidney injury in this patient.

A low serum C3 level and normal C4 level with glomerulonephritis and TMA suggested the necessity to consider the differential diagnoses of complement-mediated atypical HUS and C3 glomerulopathy in this patient. Both diseases are known to develop in the background of genetic abnormalities of the complement system leading to dysregulated complement activation via an alternative pathway [22]. In our patient, however, these diseases were unlikely because the C3 level increased with time and eventually became and was maintained at a normal level, and because the direct Coombs test was transiently positive.

In conclusion, we reported a case of a patient with PSAGN complicated by TMA, whose direct Coombs test was transiently positive. In this patient, two pathogenic factors of *Streptococcus pyogenes*, i.e., NAPlr and neuraminidase, are thought to have simultaneously induced glomerulonephritis and TMA with tubulointerstitial changes, respectively.

## Data Availability

Not applicable.

## Abbreviations

PSAGN: Poststreptococcal acute glomerulonephritis
TMA: Thrombotic microangiopathy
T-antigen: Thomsen–Friedenreich antigen
HUS: Hemolytic uremic syndrome
FFPE: Formalin fixed paraffin embedded tissue
NAPlr: Nephritis-associated plasmin receptor
PAS: Periodic acid-Schiff
IF: Immunofluorescence
FITC: Fluorescein isothiocyanate
AF594: Alexa Fluor 594
G: Glomerulus
MФ: Macrophage
